# Changes in oxygen partial pressure of brain tissue in an animal model of obstructive apnea

**DOI:** 10.1186/1465-9921-11-3

**Published:** 2010-01-15

**Authors:** Isaac Almendros, Josep M Montserrat, Marta Torres, Constancio González, Daniel Navajas, Ramon Farré

**Affiliations:** 1 CIBER Enfermedades Respiratorias, Spain; 2 Laboratori de la Son, Pneumologia, Hospital Clínic-IDIBAPS, Barcelona, Spain; 3 Departamento de Bioquímica y Biología Molecular y Fisiología, Facultad de Medicina, Universidad de Valladolid, Valladolid, Spain; 4 Unitat de Biofisica i Bioenginyeria, Facultat de Medicina, Universitat de Barcelona - IDIBAPS, Barcelona, Spain; 5 Institut de Bioenginyeria de Catalunya, Barcelona, Spain

## Abstract

**Background:**

Cognitive impairment is one of the main consequences of obstructive sleep apnea (OSA) and is usually attributed in part to the oxidative stress caused by intermittent hypoxia in cerebral tissues. The presence of oxygen-reactive species in the brain tissue should be produced by the deoxygenation-reoxygenation cycles which occur at tissue level during recurrent apneic events. However, how changes in arterial blood oxygen saturation (SpO_2_) during repetitive apneas translate into oxygen partial pressure (PtO_2_) in brain tissue has not been studied. The objective of this study was to assess whether brain tissue is partially protected from intermittently occurring interruption of O_2 _supply during recurrent swings in arterial SpO_2 _in an animal model of OSA.

**Methods:**

Twenty-four male Sprague-Dawley rats (300-350 g) were used. Sixteen rats were anesthetized and non-invasively subjected to recurrent obstructive apneas: 60 apneas/h, 15 s each, for 1 h. A control group of 8 rats was instrumented but not subjected to obstructive apneas. PtO_2 _in the cerebral cortex was measured using a fast-response oxygen microelectrode. SpO_2 _was measured by pulse oximetry. The time dependence of arterial SpO_2 _and brain tissue PtO_2 _was carried out by Friedman repeated measures ANOVA.

**Results:**

Arterial SpO_2 _showed a stable periodic pattern (no significant changes in maximum [95.5 ± 0.5%; m ± SE] and minimum values [83.9 ± 1.3%]). By contrast, brain tissue PtO_2 _exhibited a different pattern from that of arterial SpO_2_. The minimum cerebral cortex PtO_2 _computed during the first apnea (29.6 ± 2.4 mmHg) was significantly lower than baseline PtO_2 _(39.7 ± 2.9 mmHg; p = 0.011). In contrast to SpO_2_, the minimum and maximum values of PtO_2 _gradually increased (p < 0.001) over the course of the 60 min studied. After 60 min, the maximum (51.9 ± 3.9 mmHg) and minimum (43.7 ± 3.8 mmHg) values of PtO_2 _were significantly greater relative to baseline and the first apnea dip, respectively.

**Conclusions:**

These data suggest that the cerebral cortex is partially protected from intermittently occurring interruption of O_2 _supply induced by obstructive apneas mimicking OSA.

## Background

Obstructive sleep apnea (OSA) is a very prevalent disease caused by recurrent events of upper airway obstruction. One of the main physiological consequences of the ventilation disruptions associated with obstructive apneas is that the patient experiences events of repetitive oxygen desaturation in arterial blood during sleep. Recurrent hypoxemia - occurring once per minute or even more frequently in patients with severe OSA - induces oxidative stress, which has been related to the inflammation, metabolic syndrome and cardiovascular risk observed in patients with this sleep breathing disorder [1,2]. Cognitive impairment is one of the well-known consequences of OSA [3]; this is usually attributed to the oxidative stress caused by intermittent hypoxia in cerebral tissues [4-6].

The mechanisms of oxidative stress genesis in OSA are not known, but it is commonly accepted that the deoxygenation-reoxygenation cycles produce swings in the genesis of oxygen-reactive species, as in the case of the ischemia-reperfusion processes [7,8]. Thus, a close relationship between oscillations in tissue oxygen partial pressure (PtO_2_) and the magnitude of the oxidative stress during recurrent obstructive apneas should be expected in any given tissue. There are no data, however, for PtO_2 _values in brain tissue, which is the variable that most directly quantifies the oxygen available to brain cells. Although the changes induced in arterial oxygen saturation (SpO_2_) by recurrent apneas can be measured by pulse oximetry, we do not know how changes in arterial SpO_2 _translate into PtO_2 _in brain tissue. In fact, the relationship between these two variables could be modulated by rapid changes in cerebrovascular hemodynamics in response to hypoxia and hypercapnia [9-11]. It could therefore be possible that a physiological response in the cerebral hemodynamics compensates, at least in part, for the reduction in arterial SpO_2 _experienced during repeated obstructive events in OSA.

Accordingly, the aim of this work was to test the hypothesis that brain tissue PtO_2 _is partially protected from intermittently occurring interruption of O_2 _supply during recurrent arterial blood oxygen desaturations. Given the invasiveness required to make direct measurements of PtO_2 _in the brain cortex, the study was carried out in an animal model that mimicked the obstructive apneas characteristic of patients with OSA.

## Methods

### Animals

This study, which was approved by the Ethics Committee of Animal Experimentation of the University of Barcelona, was carried out on 24 Sprague-Dawley male rats (300-350 g). The animals were anesthetized intraperitoneally with Urethane 10% (1 g/Kg) and at the end of the study they were sacrificed by ex-sanguination.

### Measurement of O_2 _partial pressure in brain tissue

The head of the anesthetized rat was immobilized in prone position and a small orifice was carefully made in the dura to expose the cerebral cortex. A modified Clark's polarographic fast-response oxygen micro-electrode pipette (OX-50, Unisense A/S, Denmark; 50 μm diameter, 90% response time <2 s) was used to measure cerebral cortex PtO_2_. The oxygen micro-electrode was connected to an amplified picoammeter (Unisense A/S, Denmark) and was calibrated before each experiment in water at 100% and 21% and oxygen-free solution (NaOH 0.1 M, sodium ascorbate 0.1 M) (MicOX software, Unisense A/S, Denmark). The oxygen sensor, mounted on a micrometric positioner, was inserted vertically 2 mm from the surface of the cerebral cortex and was then slightly retracted (≈ 0.5 mm) to a point where a stable value of PtO_2_was attained within the 30-50 mmHg range. A pulse oxymeter was positioned in the rat leg to measure arterial blood saturation (504; Critical Care Systems, Inc., Waukesha, WI). The arterial SpO_2 _and brain tissue PtO_2 _signals were sampled at 60 Hz and stored for subsequent analysis.

### Application of obstructive apneas

A previously described method was used to subject the rats to a pattern of non-invasive recurrent obstructive apneas: 60 apneas/h lasting 15 s each for a total time of 1 h [12]. This model was based on a nasal mask with two tubes, one open to the atmosphere and the other connected to an airflow source to avoid rebreathing. To generate controlled obstructive apneas, two electronically synchronized electrovalves positioned in each tube were closed (airway obstruction) or opened (spontaneous breathing). Sixteen rats were subjected to obstructive apneas and another eight rats, used as controls, were identically instrumented but the valve-system in the nasal mask did not apply airway obstructions.

### Data processing

Both arterial SpO_2 _and cerebral cortex PtO_2 _were measured at baseline and at the minimum and maximum attained during the first ten apneas and during the following apneas every 10 min up to 1 h. Results are shown as mean ± SE. The time dependence of arterial SpO_2 _and brain tissue PtO_2 _was carried out by Friedman repeated measures ANOVA by comparing the sample points at the first apnea and every 10 min up to 1 h with respect to the steady state at the beginning of the experiment. Comparisons were undertaken by means of the Student-Newman-Keuls method, where appropriate.

## Results

**Figure 1:**
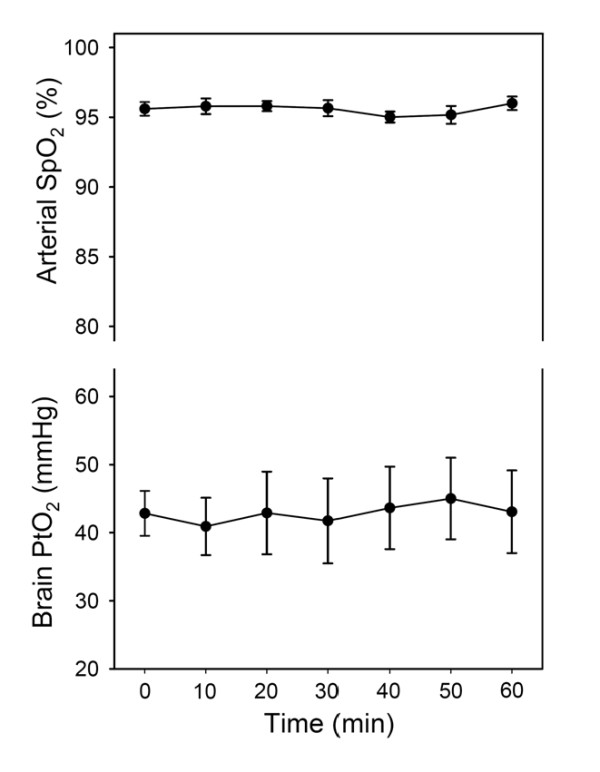
**Time course of the values of arterial oxygen saturation (SpO_2_) and brain tissue O_2 _partial pressure (PtO_2_) recorded in control rats**. No changes were found in SpO_2 _and PtO_2 _values over the 60 min of the experiment. Results are shown as mean ± SE (n = 8). The time dependence of arterial SpO_2 _and brain tissue PtO_2 _was assessed with a Friedman repeated measures ANOVA by comparing sample points at the first apnea and during the apneas at minute 10 and every 10 min up to 1 h with respect to baseline.

As expected, no significant changes were observed in the control group in either SpO_2 _(95.6 ± 0.5 vs 96.0 ± 0.5%) or PtO_2 _(42.8 ± 3.3 vs 43.1 ± 6.1 mmHg) between the baseline and after 60 min of spontaneous breathing with no apneas, respectively (Fig. 1).

**Figure 2:**
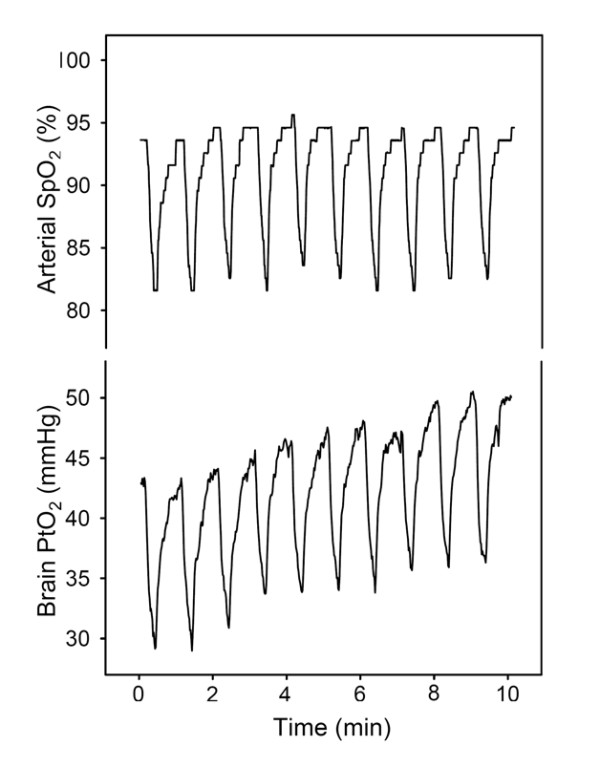
**Example of oxygen arterial saturation (SpO_2_) and brain tissue O_2 _partial pressure (PtO_2_) signals recorded during the first ten minutes from a rat subjected to recurrent obstructive apneas**. The SpO_2 _dips, mimicking those observed in OSA patients, were paralleled by similar dips in PtO_2_. However, while maximum and minimum values in SpO_2 _showed a stable pattern (top), PtO_2 _exhibited a progressive tendency to increase (bottom).

Figure 2 shows an example of the data recorded during the first 10 min after application of recurrent apneas in a representative rat. Arterial SpO_2 _exhibited a stable recurrent pattern (Fig. 2, top). Each apnea was reflected by a fast decrease in SpO_2 _followed by a recovery, with minimum and maximum values typical of patients with OSA [13]. Brain tissue PtO_2 _followed a periodic time course similar to that of arterial SpO_2_, but a trend toward increasing PtO_2 _was apparent (Fig. 2, bottom).

**Figure 3:**
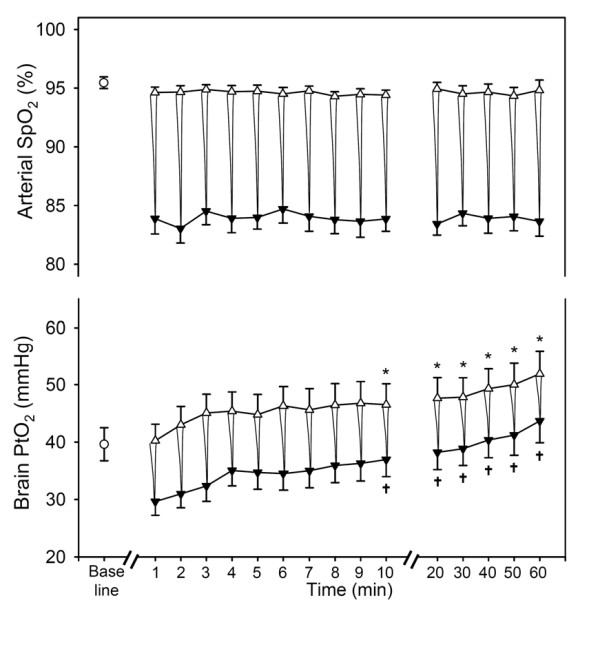
**Time course of the maximum (Δ) and minimum (▼) values of arterial oxygen saturation (SpO_2_) and brain tissue O_2 _partial pressure (PtO_2_) recorded during each obstructive apnea**. A schematic representation of the corresponding dips in both variables is superimposed for the purpose of clarity. Baseline value is represented by an open circle. Maximum and minimum values of PtO_2 _experienced a time-dependent increase (both, p < 0.001). In contrast, no changes were found in SpO_2 _maximum and minimum values. Results are shown as mean ± SE (n = 16). The time dependence of arterial SpO_2 _and brain tissue PtO_2 _was assessed with Friedman repeated measures ANOVA by comparing sample points at the first apnea and during the apneas at minute 10 and every 10 min up to 1 h with respect to baseline for maximum values or first event for minimum values. Comparisons were undertaken by means of the Student-Newman-Keuls method. * p < 0.05 respect to baseline ✟ p < 0.05 respect to the first apneic event.

Figure 3 shows the average data obtained in the group of 16 rats subjected to recurrent apneas for 60 min. Arterial SpO_2 _showed a stable periodic pattern (no changes in maximum and minimum values). There was no significant difference between the baseline and the maximum values attained after recovery from each apnea. Moreover, no significant differences were found between the minimum values in arterial SpO_2 _attained during the different apneas. By contrast, brain tissue PtO_2 _exhibited a different pattern from that of arterial SpO_2 _(Fig. 3, bottom). During the first apnea (at time 1 min), PtO_2 _was considerably reduced to a minimum value of 29.6 ± 2.4 mmHg (range 13.5 - 47.7 mmHg) from baseline (39.7 ± 2.9 mmHg) (range 25.5 - 64.0 mmHg). In the following apneas, minimum and maximum values of PtO_2 _progressively increase (both, p < 0.001). After the first 10 min of repeated airway obstructions, the maximum value attained by PtO_2 _after the apnea (46.5 ± 3.7 mmHg) (range 28.2 - 76.5 mmHg) was significantly greater (p < 0.05) than baseline and this difference increased further throughout the experiment (PtO_2 _after the apnea was 51.9 ± 3.9 mmHg at 60 min) (range 34.5 - 84.9 mmHg). The minimum values attained by PtO_2 _in each apnea followed a pattern similar to that of the maximum values: from 29.6 ± 2.4 mmHg (range 13.5-47.7 mmHg) during the first apnea to 43.7 ± 3.8 mmHg (range 29.1-83.1 mmHg) at 1 h (p < 0.05). The magnitude of the PtO_2 _swings showed no significant dependence on time over the course of the experiment.

## Discussion

We found that cerebral tissue PtO_2 _followed a pattern of changes markedly different from that of arterial SpO_2 _when the animals were subjected to recurrent obstructive apneas mimicking OSA (Fig. 3). Indeed, during the first obstructive apnea there was an immediate transient reduction in the oxygen partial pressure in brain tissue; this was quickly restored during reoxygenation. In contrast to SpO_2_, the maximum and minimum values observed in PtO_2 _during the following apneas experienced an increase with respect to the basals, indicating that the brain was partially protected from the intermittent interruption of O_2 _supply during recurrent arterial oxygen desaturations.

Although changes in the PtO_2 _of several tissues were previously measured in models of hypoxia, endotoxemia and hemorrhage [14-18], no data describing how brain tissue PtO_2 _is affected by airway obstructions mimicking OSA were available before the present study. To carry out this work we used a previously described non-invasive animal model [12] specifically designed to apply recurrent obstructive apneas in a stable and controlled manner, free from confounding factors caused by the comorbidities that typically accompany OSA. The fact that the animals in this model were anesthetized could affect the systemic cardio-respiratory response to occlusions when compared with non-anesthetized animals. Accordingly, the response to recurrent obstructive apneas in terms of resulting arterial SaO_2 _could be slightly different in anesthetized and non-anesthetized animals. However, the SaO_2 _signals recorded in our anesthetized rats concur to a reasonable extent with the ones observed in OSA patients experiencing a pattern of obstructive apneas similar to the one applied to our rats (60 apneas/h lasting 15 s each). In contrast with animal models in which hypoxia/reoxygenation was applied by periodically changing the O_2 _concentration of the breathed air, the model used in this study applied actual obstructive apneas [19]. Accordingly, this model was able to mimic the respiratory events in OSA patients more realistically. Indeed, in addition to intermittent hypoxia, the animal experienced increased inspiratory efforts to breathe against a periodically obstructed airway and was exposed to the recurrent hypercapnia associated with obstructive apneas. This is particularly relevant here since these two stimuli have potential systemic and cerebral hemodynamic effects [10,20] that could alter the relationship between arterial SpO_2 _and brain tissue PtO_2_.

Given the heterogeneity in brain tissue oxygen tension [21], in order to measure the time course of cerebral cortex PtO_2 _we focused our attention on tissue places with baseline values within the range of 30-50 mmHg, which are the most representative values observed in the majority of tissues (including the brain) during normoxia [22,23]. The modified Clark's micro-electrode oxygen sensor is particularly suited to the measurement of brain PtO_2 _[10,14,24-26]. When compared with techniques requiring chronic implantation of paramagnetic oxygen-sensitive material in the tissue under study (electron paramagnetic resonance technique) [22], a micro-electrode is minimally invasive and induces minimal tissue trauma [27], as reflected by the stable PtO_2 _values obtained in the control rats. One advantage of the micro-electrode technique is that its small size (50 μm) allows the measurement of brain tissue PtO_2 _at a micro-regional level, in contrast to the near-infrared spectroscopic technique, which explores a greater tissue volume and may therefore include large blood vessels [28,29]. One important feature of the specific micro-electrode oxygen sensor used in this work is that its rapid response time (90% response in less than 2 s) allowed us to accurately determine the rapid changes induced in PtO_2 _during recurrent obstructive apneas.

The gradual increase in brain tissue PtO_2 _was significant within the first 10 apneic episodes (10 min) (Fig. 3). This response time falls within the time scale of previously reported cerebrovascular and physiological oxygenation responses to ventilatory challenges. In fact, it was reported that cerebral blood flow increased almost immediately after the application of acute isocapnic hypoxia to healthy volunteers [11]. Moreover, the low values of brain PtO_2 _induced by hemorrhage in a swine model (PtO_2 _decreased from 40 mmHg at baseline to 5 mmHg) were recovered in only ≈ 10 min [14], while brain PtO_2 _in rats exhibited a fast response when subjected to changes in the concentration of O_2 _and CO_2 _in inspired air; the increase in PtO_2 _was also CO_2 _dose-dependent [10]. In this respect, it should be stressed that our model would produce an increase in blood PCO_2 _concomitant with oxygen desaturation. Hypercapnia may amplify the effects of hypoxemia on brain blood flow, thereby attenuating the impact of systemic hypoxia on PtO_2 _drops in the brain [30]. Nevertheless, it should be mentioned that prolonged hypercapnia tends to blunt the vasodilatating effect of CO_2 _[31,32]. Although, to our knowledge, there are no data relating the prolonged intermittent hypercapnia found in OSA to CO_2 _regulation of cerebral blood flow, alterations in the cerebral perfusion of OSA patients are well documented [33]. Interestingly, the potential effect of hypercapnia modulating the cerebral flow and the mechanical stimuli characterizing obstructive apneas could be tested in future studies by subjecting animals to intermittent hypoxia without any airway obstructions.

The observed response was only partially effective in protecting brain tissue from deoxygenation-reoxygenation cycling. Indeed, despite the fact that brain PtO_2 _showed a tendency to increase, PtO_2 _swings were still found in brain tissue (Fig. 3), potentially explaining the increase in reactive oxygen species (or the oxidative stress damage) encountered in brain tissue by other authors [4,5]. At the outset, it would appear that the magnitude of PtO_2 _decrease in the desaturation valleys is not enough to trigger an ischemia-like process. With the PtO_2 _recorded in our experiments an alteration in electron transport with subsequent energetic derangement and loss of cell ion gradients should not be expected, owing to the high O_2 _affinity of cytochrome oxidase [34,35]. Similarly, an anomalous activation of reactive oxygen species generating enzymes in the cell cytoplasm is not conceivable as there is no basis to the suspicion of alterations in intracellular Ca^2+ ^homeostasis or exhaustion of cell NAD^+ ^in the reoxygenation phase of the obstruction [36,37]. Yet, as mentioned above, it is possible that the protective effect of hypercapnia via an increase in cerebral blood flow may progressively decline with the duration of OSA, and hemoglobin desaturation could therefore be directly transmitted to brain tissue PO_2_: i.e., in the long run a certain level of brain hypoxia, and thereby of oxidative stress, may occur. Another potential factor in the genesis of reactive oxygen species in this situation is inflammation. It is well documented that OSA patients and intermittent hypoxia models have sensitized leukocytes [38,39] that produce higher amounts of reactive oxygen species at rest and under stimulation, and an increased expression of inflammatory cytokines and soluble adhesion molecules [40]. Consistent with this suggestion, it has been reported that short episodes of hypoxia (a total of 12 apneas of 30 s each) cause a dramatic increase in the number of leukocytes adhering to brain microvessels [41,42]. Thus, hypoxemia-induced leukostasis in brain microcirculation and sensitization of leukocytes can satisfactorily explain the genesis of oxidative stress, despite the maintenance of PtO_2_. This oxidative stress in turn can be pathogenic to the cognitive deterioration (and other neurological alterations) observed in both animal models [4,5] and patients with OSA [3]. It is worth pointing out, in this respect, that intermittent hypoxia, such as the one detected regardless of a trend toward increasing PtO_2 _(Fig. 3), is more deleterious, for similar levels of hypoxia, than constant hypoxia [7,43].

As it is impossible to measure brain tissue PtO_2 _non-invasively in humans, the only available data on brain oxygenation in patients with OSA were obtained by measuring indirect variables. For instance, blood flow in cerebral artery measured by transcranial Doppler [44,45] or brain tissue total hemoglobin and oxyhemoglobine measured by near-infrared spectroscopy [28,29]. These findings showed that the values observed by these indirect measurements of brain oxygenation were lower in patients with sleep disorder breathing than in healthy persons. The differences between groups could be masked, however, by confounding factors such as age and obesity, which have been shown to modify cerebral hemodynamics [46-49] and cerebrovascular autoregulation in patients with OSA [45]. Interestingly, the application of the methodological approach devised in the present work to hypertensive, obese, diabetic or old rats could be useful for investigating how comorbidities typically found in OSA interact with physiological mechanisms to partially protect brain tissue from recurrent apneas.

## Conclusions

We found that the oxygen partial pressure in the brain tissue of rats subjected to recurrent obstructive apneas mimicking OSA is partially protected from intermittent interruption of O_2 _supply during dips in oxygen desaturation found in arterial blood. This phenomenon, whose effectiveness could be reduced by common comorbidities, could play a role in modulating the neurocognitive consequences of OSA.

## Competing interests

The authors declare that they have no competing interests.

## Authors' contributions

The conception and scientific direction of this work was undertaken by RF. Animal experimentation was carried out by IA and MT. Data processing and statistical analysis was undertaken by IA, JMM and RF. IA, JMM, RF, DN and CG participated in the discussion of the results and contributed to the manuscript draft. All authors read and gave critical input to this manuscript.
